# Antibacterial Activity of AI-Hemocidin 2, a Novel N-Terminal Peptide of Hemoglobin Purified from *Arca inflata*

**DOI:** 10.3390/md15070205

**Published:** 2017-06-29

**Authors:** Chunlei Li, Jianhua Zhu, Yanqing Wang, Yuyan Chen, Liyan Song, Weiming Zheng, Jingjing Li, Rongmin Yu

**Affiliations:** 1 Biotechnological Institute of Chinese Materia Medica, Jinan University, Guangzhou 510632, China; lcl992@126.com (C.L.); tzhujh@jnu.edu.cn (J.Z.); 13533055513@163.com (Y.C.); 2 National Engineering Research Center of Microsphere Technology for Controlled-Release Drug Delivery, Zhuhai 519090, China; wangyanqing@livzon.cn; 3 Department of Pharmacology, Jinan University, Guangzhou 510632, China; tsly@jnu.edu.cn (L.S.); miguelcheng170629@gmail.com (W.Z.); 13422016695@126.com (J.L.)

**Keywords:** *Arca inflata*, peptide, hemoglobin, purification, structural elucidation, antibacterial activity

## Abstract

The continued emergence of antibiotic resistant bacteria in recent years is of great concern. The search for new classes of antibacterial agents has expanded to non-traditional sources such as shellfish. An antibacterial subunit of hemoglobin (Hb-I) was purified from the mantle of *Arca inflata* by phosphate extraction and ion exchange chromatography. A novel antibacterial peptide, AI-hemocidin 2, derived from Hb-I, was discovered using bioinformatics analysis. It displayed antibacterial activity across a broad spectrum of microorganisms, including several Gram-positive and Gram-negative bacteria, with minimal inhibitory concentration (MIC) values ranging from 37.5 to 300 μg/mL, and it exhibited minimal hemolytic or cytotoxic activities. The antibacterial activity of AI-hemocidin 2 was thermostable (25–100 °C) and pH resistant (pH 3–10). The cellular integrity was determined by flow cytometry. AI-hemocidin 2 was capable of permeating the cellular membrane. Changes in the cell morphology were observed with a scanning electron microscope. Circular dichroism spectra suggested that AI-hemocidin 2 formed an α-helix structure in the membrane mimetic environment. The results indicated that the anti-bacterial mechanism for AI-hemocidin 2 occurred through disrupting the cell membrane. AI-hemocidin 2 might be a potential candidate for tackling antibiotic resistant bacteria.

## 1. Introduction

The emergence and rapid spread of bacterial infections caused by antibiotic resistant strains has led to a health crisis in recent years [1,2,3,4,5,6]. The threat of drug-resistant bacterial infections has been attributed to the abuse and misuse of antibiotics as well as a lack of development of new antibacterial agents [1,3,5,6]. A recent investigation demonstrated that premature deaths caused by antibiotic resistant bacteria will rise to 1,000,000 lives per year and cost the global economy $100 trillion by 2050 [7]. The urgent need for new types of antibacterial agents has led to innovative multidisciplinary research.

Antimicrobial peptides (AMPs) are produced by hosts as part of the innate immune response and constitute the first line of defense against invading pathogenic microbes [8]. AMPs typically comprise 10–50 amino acids, including basic residues such as arginine or lysine, that possess a positive net charge and amphipathic secondary structure such as an α-helix or β-sheet [9,10,11]. Based on these properties, AMPs target and perturb the microbial cytoplasmic membranes that typically present anionic surfaces via an ionic interaction. Some investigations have demonstrated that most AMPs permeabilize microbial cytoplasmic membranes. Although most AMPs affect the integrity of microbial membranes, it remains to be determined whether the microbial membrane is the only site of action or whether membrane permeabilization is always fatal. Some AMPs that do not extensively permeabilize microbial membranes might interact with intracellular components and thus affect microbe viability. The mechanism of action of AMPs remains to be fully elucidated [12]. Compared with antibiotics, AMPs have drawn increasing attention as novel antibacterial agents to combat bacterial invasion due to their special mechanisms of action, non-inducible bacterial resistance and lack of detrimental effects on humans [13]. AMPs derived from marine organisms are promising candidates for drug development and as nutritional food additives. By contrast with on-land compounds, marine peptides have diverse sequences, unique structures and prominent bioactivities as a result of particular adaptations to oceanic environments [14]. Many peptides and derivatives (such as mytilin, defensin, mytimycin and myticins) derived from shellfish have been reported to exhibit antimicrobial activity [15] and have potential applications in the healthcare and fishery industries [16,17].

Hemoglobin, which is widely distributed among organisms, has previously been regarded to function exclusively as an endogenous respiratory protein. However, studies have shown that hemoglobin exerts inhibitory effects on bacteria and is a source of antibacterial peptides, in addition to its role as an oxygen carrier, and its involvement in transporting sulfide and regulating the acid–base balance [18]. Antibacterial peptide fragments derived from hemoglobin subunits (hemocidins) have been considered as important effectors of the innate immune response against pathogen infections [19]. Hemocidins in humans, bovine and channel catfish were found to possess potent antimicrobial activities against pathogenic bacteria [20,21,22,23,24]. P3, a novel hemoglobin-related peptide derived from bovine erythrocytes, exhibited significant antimicrobial activity [25]. In addition, Hb137-141, a peptide isolated from the enzyme digestion of bovine hemoglobin alpha-chain showed potent antibacterial activity against *Escherichia coli*, *Salmonella enteritidis*, *Listeria innocua*, *Micrococcus luteus* and *Staphylococcus aureus* [26]. Although studies on hemocidins have been performed in a wide range of species, they have been limited to vertebrates. In some invertebrates, such as arthropods and mollusks, hemolymph is analogous to the blood in vertebrates. There are three types of oxygen-transport proteins in the hemolymph of invertebrates: hemoglobin, hemocyanin and hemerythrin [27]. Among these, hemocyanins are regarded as the major, multifunctional proteins in invertebrate hemolymph, responsible for oxygen transport and contributing to innate immunity in resisting invasion by pathogenic microbes [27,28]. Recently, hemocyanin-derived antimicrobial peptides have been identified from abalone, a marine mollusk [28]. However, few studies have focused on the hemoglobins and hemocidins of invertebrates.

*A. inflata* is a special type of bivalve shellfish that contains hemoglobin. *A. inflata*, which belongs to the Arcidae family under the phylum Mollusca and the class Lamellibranchiata, is widely distributed in the western Pacific Ocean. It has been used as a nutritious food material around the world, and a traditional medicine for treating blood deficiency, epigastric pain and indigestion for centuries in China. A polypeptide from *A. inflata* with impressive antitumor activity was reported by our research group [29]. However, little attention has been devoted to antibacterial peptides in *A. inflata.* As a result of their lack of a specific immune system, invertebrates such as *A. inflata* rely on the innate immune response comprising hemocytes and humoral factors to resist invasion from pathogens in the environment [30]. Therefore, it may be possible to discover novel antibacterial agents in marine invertebrates such as *A. inflata*.

Enzymatic hydrolysis is a feasible method with which to discover potent antibacterial peptides. Peptides hydrolyzed by trypsin, preferably containing arginine and lysine, usually possess positive charges and exhibit antibacterial activity [31,32]. However, it is difficult to identify the bioactive peptides among the huge number of peptides produced by hydrolysis. In addition, the bioactive peptides may become inactive during the enzymatic hydrolysis process. Since bioactive peptides possessing similar domain and physiochemical parameters exhibit similar bioactivities, bioinformatic analysis is a rapid and convenient method for discovering novel bioactive peptide sequences. Hence, enzymatic hydrolysis simulation combined with bioinformatic analysis is an efficient and innovative approach to discover potential antibacterial agents.

The aim of this study was to purify and characterize antibacterial agents from *A. inflata.* A novel antibacterial peptide, AI-hemocidin 2, was discovered from *A. inflata* and the physicochemical and structural properties were determined. Moreover, the preliminary mechanism of antibacterial action of AI-hemocidin 2 was revealed.

## 2. Results and Discussion

### 2.1. Purification of Hb-I

**Figure 1:**
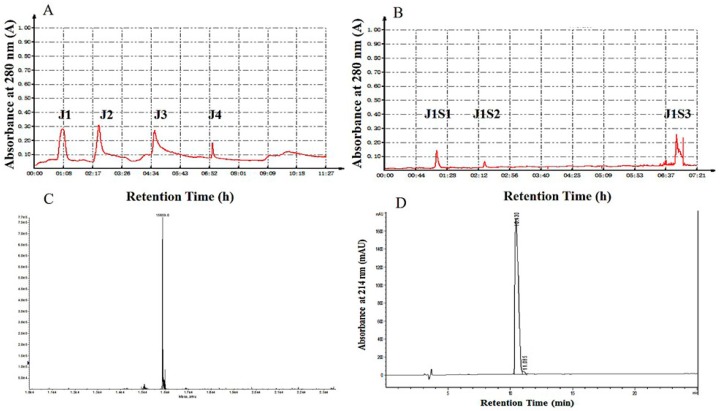
Purification of hemoglobin Hb-I: (**A**) separation of active fractions in the supernatant by DEAE Fast Flow anion exchange chromatography; (**B**) purification of active fraction J1S1 which contained Hb-I obtained from (**A**) by SP Fast Flow anion exchange chromatography; (**C**) ESI-MS mass spectrum of Hb-I; and (**D**) the final purification of Hb-I.

Following an antibacterial assay, Hb-I was purified from the mantles of *A. inflata* by ammonium sulfate precipitation and column chromatography. The results of the purification process are shown in Figure 1 and Table 1.

The highest antimicrobial activity peak, J1S1, was obtained by a two-step purification procedure of ion-exchange chromatography (Figure 1A,B). J1S1 exhibited the highest antimicrobial activity against *E. coli* with inhibition zone diameters of 16.19 ± 0.62 mm. To evaluate the purity of fraction J1S1, tricine sodium dodecyl sulfate polyacrylamide gel electrophoresis (Tricine-SDS-PAGE), native polyacrylamide gel electrophoresis (Native-PAGE) and RP-HPLC were applied, as described in a previous study [33]. A single band in both Tricine-SDS-PAGE and Native-PAGE revealed that J1S1 was a pure polypeptide with an approximate molecular weight of 15 kDa (Figure S1A,B). Furthermore, J1S1 was loaded onto a reversed-phase C18 column to confirm the purity. A single peak was found at 10.43 min (Figure 1D), highlighting the high purity of J1S1. Electrospray-ionization mass spectrometry (ESI-MS) analysis of purified J1S1 revealed that its accurate molecular weight was 15,859.0 Da (Figure 1C). Compared with organic solvent extraction, ammonium sulfate precipitation can be used to obtain more proteins and peptides with bioactivity. Many studies have used ammonium sulfate precipitation for antibacterial peptide isolation, including the purification of plantaricin 163 [34], plantaricin A-1 [35] and sakacin LSJ618 [36]. *E. coli* is one of the most common pathogenic bacteria and a frequent cause of nosocomial and community-acquired bacterial infections. In recent years, infections with *E. coli* have been increasingly reported, and the number of *E. coli* isolates found to be resistant to antibiotics, especially to cephalosporins and fluoroquinolones, has increased significantly during the last two decades [37]. The polypeptide fraction J1S1, purified from *A. inflata*, which is capable of inhibiting *E. coli*, is a promising candidate for the development of antibacterial agents.

### 2.2. Identification of Hb-I

**Figure 2:**
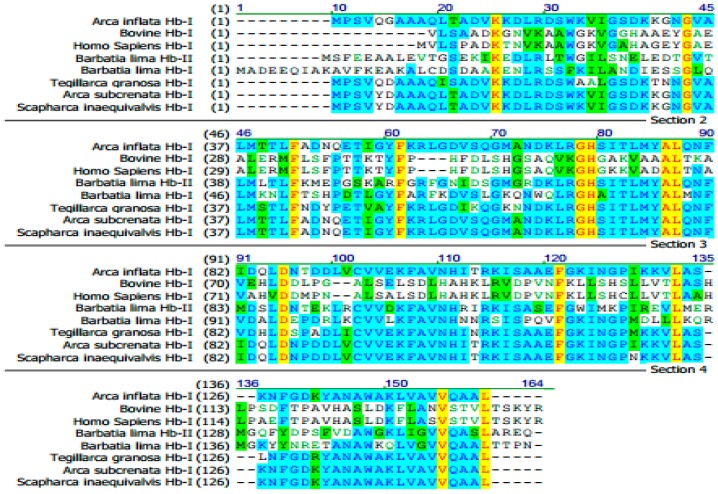
Sequence comparison of Hb-I with the hemoglobin from other invertebrates. Identical amino acid residues are highlighted in yellow, and similar amino acid residues are highlighted in blue and green, respectively.

J1S1 was subjected to amino acid sequence analysis by automated Edman degradation. The N-terminal amino acid sequence was determined to be PSVQGAAQQLTADVK (Figure S2). The results of BLAST searches showed only one match to the hemoglobin subunit I (Hb-I) of *Scapharca broughtonii* (BAM63323.1) with a high degree of similarity (93%). With the polymerase chain reaction (PCR) technique, J1S1 that contained an open reading frame of 441 nucleotides was found to be identical to Hb-I in *A. inflata* (GenBank: AB713934.1). By BLAST search, Hb-I in *A. inflata* showed high similarity with the hemoglobin subunit I of *Scapharca inaequivalvis*, *Arca subcrenata* and *Tegillarca granosa*, but little similarity with the hemoglobin-related antibacterial peptides of vertebrates (Figure 2). Some studies have demonstrated clear antibacterial activity in the hemoglobin of vertebrates [19,20,21,22,23,24,25,26,27]. However, there has been little focus on the antibacterial effects of invertebrate hemoglobin and hemocidins. Compared with the hemoglobin subunit, hemocidins have stronger antibacterial activity and stability [38,39] and therefore have the potential to be potent antibacterial agents.

### 2.3. Bioinformatic Analysis and Antibacterial Function Screening

**Figure 3:**
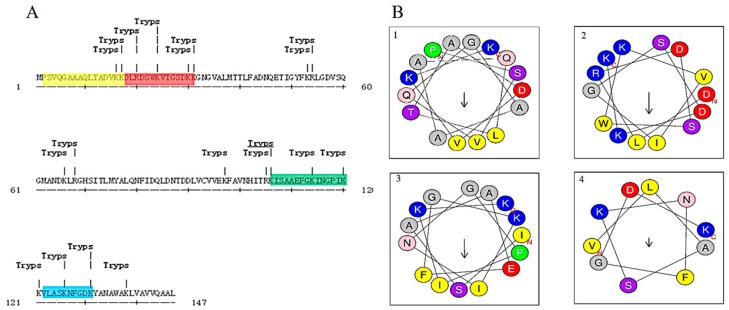
In silico analysis of Hb-I: (**A**) Trypsin hydrolytic sites and peptide fragments aligned with APD; and (**B**) Schiffer and Edmundson helical wheel projection of AI-hemocidins. The hydrophobic residues are yellow, positively charged hydrophilic residues are blue, negatively charged hydrophilic residues are red, asparagine and glutamine are in pink color, small neutral residues are gray, and small polar residues are purple.

Protein hydrolysis by enzymes is a feasible way to discover potent antibacterial peptides hidden in proteins. Peptides hydrolyzed by trypsin, preferably containing arginine and lysine, usually possess positive charges and exhibit antibacterial activity [31,32]. For example, indolicidin, which contained arginine or lysine at its C-terminus, displayed potent antibacterial activity [40]. Hydrolysis simulation combined with bioinformatic analysis is an efficient and innovative approach to discover potential antibacterial agents. Through trypsin hydrolysis simulation and bioinformatic analysis using the antimicrobial peptide database (APD), AI-hemocidins, including several peptide fragments with potential antibacterial function, were identified (Figure 3A).

The sequences and physicochemical properties of AI-hemocidins are listed in Table 2. All peptides had a net positive charge and were proposed to be cationic peptides. The ratios of polar residues to nonpolar residues were approximately 50%. Among them, AI-hemocidin 2 showed the highest ratio of polar residues to nonpolar residues (71.43%), whereas AI-hemocidin 2 had the lowest hydrophobicity of 0.043. The predicted helix structures of the peptides are shown in Figure 3B. All peptides could form helixes, though only AI-hemocidins 2 was capable of forming a stable amphipathic structure that contained a hydrophobic side and a positively charged surface. Generally, antibacterial peptides possess several features indicative of their antibacterial activity [41,42]. One feature is their positive charge, and another feature is the presence of at least 50% of hydrophobic residues. The positive charge is conducive to an electrostatic interaction with the bacterial membrane, which is negatively charged [41]. The high ratio of hydrophobic residues is thought to contribute to disruption of the bacterial membrane. However, a more crucial feature of the peptides possessing antibacterial action is the formation of an ordered and stable amphipathic structure. The hydrophobic side of the structure is thought to enter into and disrupt the bacteria membrane, whereas the hydrophilic residues are considered to provide the opportunity to combine or adhere to the membrane surface [42]. Although AI-hemocidin 2 contained a high ratio of polar residues and low hydrophobicity, the amphipathic structure that formed between the hydrophobic side and a positively charged surface conferred antibacterial activity.

The antibacterial activities of Hb-I and AI-hemocidins are shown in Table 3. Hb-I demonstrated adequate antibacterial activity against *E. coli* and *S. aureus*. Among the AI-hemocidins, AI-hemocidin 2 showed the high activity against the test strains, with the exception of *Enterococcus faecalis*. *S. aureus* exhibited the highest sensitivity to AI-hemocidin 2 with a MIC value of 37.5 μg/mL (22.77 μM). Compared with Hb-I, the antibacterial effect of AI-hemocidin 2 was enhanced. Although Hb-I was initially screened with *E. coli*, the antibacterial activity of AI-hemocidin 2 against several Gram-positive bacteria was observed. The results suggested that AI-hemocidin 2 was an antibacterial peptide with a broad antimicrobial spectrum. In addition, AI-hemocidin 1 displayed antibacterial activity selectively against Gram-negative bacteria *Pseudomonas aeruginosa* and *E. coli* with a MIC value of 75 μg/mL (47.35 μM). Peptides derived from the erythrocytes of various living organisms have also been reported to exhibit antibacterial activity. Recently, hydrolyte fractions of bovine hemoglobin were reported to have antibacterial activity against bacteria invasion. A novel hemoglobin peptide, P3 derived from bovine erythrocytes, assumed an α-helical conformation and exhibited modest antimicrobial activity in vitro. P3 killed bacteria rapidly by disrupting the bacterial cytoplasmic membrane and disturbing the intracellular calcium balance. Hemoglobin-related peptides are thereby becoming a new source of antibacterial agents that are capable of inhibiting pathogenic bacteria.

### 2.4. Circular Dichroism Spectra and 3D Secondary Structure Prediction

**Figure 4:**
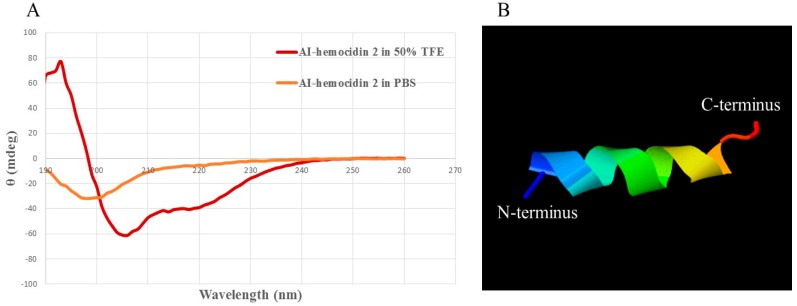
Secondary structure characterization of AI-hemocidin 2: (**A**) circular dichroism (CD) spectra of AI-hemocidin 2; and (**B**) 3D structural prediction of AI-hemocidin 2. The colors are meaningless and only used to perform the secondary structure in a nice-looking way.

The secondary structure of AI-hemocidin 2 was investigated in different environments, including 10 mM PBS solution and 50% trifluoroethanol solution by circular dichroism (CD) spectroscopy [43]. As illustrated in Figure 4A, peptides displayed random coil conformations in PBS. However, the spectra of AI-hemocidin 2 featured an α-helix conformation in the presence of 50% TFE, which was indicated by the presence of two negative dichroic bands at 208 and 222 nm [44]. The results of the CD spectrum in AI-hemocidin 2 were accordant to the secondary structure predicted by the helical wheel projection (Figure 3B). The modeling structure of AI-hemocidin 2 was proposed by Iterative Threading ASSEmbly Refinement (ITASSER) (Figure 4B). The structure of the antibacterial peptides allows for their insertion into bacterial membranes forming a voltage-dependent ion channel, thereafter altering the permeability of the bacterial cell, leading to disruption of cytoplasmic processes and target cell lysis [11]. The high level of α-helix in AI-hemocidin 2 contributed to the sensitive antibacterial activity.

### 2.5. Assays for Hemolysis and Cytotoxicity

**Figure 5:**
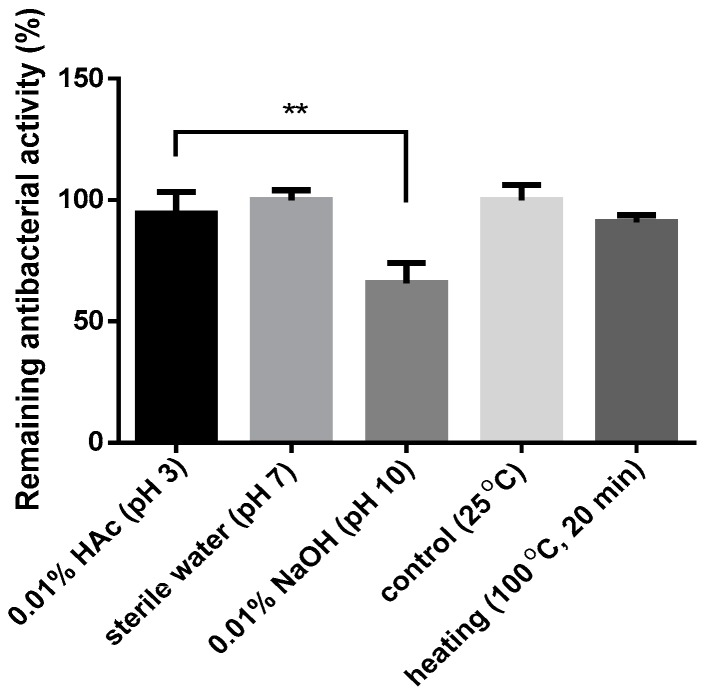
Stability of the antibacterial activity of AI-hemocidin 2 against *E. coli* during changes in pH and heat, ** *p* < 0.05.

AI-hemocidin 2 showed low hemolytic activity on murine red blood cells. The hemolysis of AI-hemocidin 2 reached a maximum of 10.27%, even at a high concentration of 500 μg/mL, which was 13 times higher than its MIC for *S. aureus* (Table 3). The HEK293 cell viability was approximately 88% after treatment with AI-hemocidin 2 at 250 μg/mL. The half maximal inhibitory concentration of cell viability was larger than 1000 μg/mL (Table 4). The cytotoxic concentrations for HEK cells were higher than the MIC values. These results indicated that AI-hemocidin 2 displayed excellent bacteria selectivity (Table 4). Piscidin 1, a peptide purified from hybrid striped bass, possesses significant antibacterial action as well as cytotoxic activity [45]. The mechanism of action of its bactericidal and fungicidal effects was operated via targeting and disrupting the bacterial and fungal membranes [46,47]. However, the application of piscidin 1 has been limited on account of its cytotoxic effects. On the contrary, hemoglobin-derived peptides exhibit antibacterial activity with low cytotoxicity. SHβAP and P3 were both hemoglobin-related antibacterial peptides derived from the liver of skipjack tuna and bovine, respectively [25,45]. SHβAP and P3 both showed low hemolytic activity and cytotoxicity, although their mechanisms of antibacterial action were similar to Piscidin 1. The finding of low cytotoxicity and low hemolytic activity of AI-hemocidin 2 is comparable to SHβAP and P3. The antimicrobial activity of AI-hemocidin 2 was not greatly affected by pH or heat treatment (Figure 5). As shown in Figure 5, compared with 0.01% HAc (pH 3) and sterile water (pH 7), the antibacterial activity of AI-hemocidin 2 was significantly decreased (*p* < 0.05) in 0.01% NaOH (pH 10), but still retained 60.76% antibacterial activity. The net charge of AI-hemocidin 2 (p.I. 8.5) was negative in 0.01% NaOH (pH 10) and this negatively charged state was not optimal for the association of AI-hemocidin 2 with the negatively charged bacterial membrane for antibacterial activity. The binding ability of AI-hemocidin 2 would have been decreased in 0.01% NaOH (pH 10), thus reducing the antibacterial activity. However, various amino acids display different isoelectric points and different charges in different solutions. AI-hemocidin 2 contained three aspartic acids at the hydrophilic side. The net charge of the hydrophilic portion with aspartic acids might be positive in 0.01% NaOH (pH 10), providing the possibility of an interaction between AI-hemocidin 2 and the negatively charged bacterial membrane. Therefore, AI-hemocidin 2 retained antibacterial activity even in 0.01% NaOH (pH 10) showing its potential as a candidate antibacterial agent.

### 2.6. Effects of AI-Hemocidin 2 on the Permeability of the Bacterial Membrane

**Figure 6:**
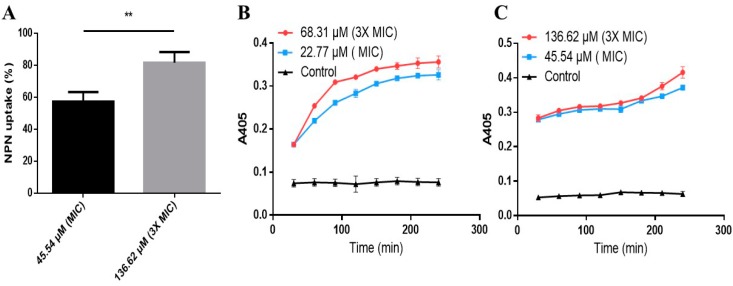
Membrane permeabilizing activity of AI-hemocidin 2: (**A**) the outer membrane permeabilization of *E. coli* by AI-hemocidin 2, NPN uptake (%) is relative to polymyxin B, a positive control with a strong outer membrane permeabilizing property, ** *p* < 0.01; (**B**) cytoplasmic membrane permeabilization of *S. aureus* by AI-hemocidin 2; and (**C**) cytoplasmic membrane permeabilization of *E. coli* by AI-hemocidin 2. Each assay was performed in triplicate, and the results are presented as the mean ± SD.

Increasing the permeability of the bacterial membrane is a possible mechanism by which peptides exert antibacterial function. Outer membrane permeabilization by AI-hemocidin 2 was assessed by an NPN uptake assay. Upon destabilization of the cellular membrane by AMPs, NPN inserted into the damaged membrane and emitted a strong fluorescence signal in the hydrophobic interior of the cell membrane [48]. As illustrated in Figure 6A, 45.54 μM (MIC) of AI-hemocidin 2 caused 57.2% NPN uptake by *E. coli* at 360 s after sample treatment, whereas 136.62 μM (3 × MIC) of AI-hemocidin 2 induced higher NPN uptake (81.66%). The results indicated that AI-hemocidin 2 was capable of permeabilizing the outer membrane in a dose-dependent manner. The permeabilization of the inner cytoplasmic membrane was measured by a β-galactosidase assay. *O*-nitrophenyl-β-d-galactopyranoside (ONPG) is hydrolyzed into o-nitrophenol in response to β-galactosidase released from the cytoplasm when the cytoplasmic membrane is permeable [49]. Therefore, ONPG was chosen as a probe to determine the permeability of the cytoplasmic membrane by spectrophotometry at 405 nm. β-galactosidase leakage was observed from *S. aureus* (Figure 6B) and *E. coli* (Figure 6C) in the presence of AI-hemocidin 2 in a dose- and time-dependent manner. There was no change over time in the control cell medium. As shown in Figure 6B,C, the absorbance values with 3 × MIC of AI-hemocidin 2 were not much higher than the values with 1 × MIC of AI-hemocidin 2. This may be because the 1 × MIC concentration permeabilized the bacterial cytoplasmic membrane to a sufficient degree and that the 3 × MIC concentration just accelerated this process. In addition, the absorbance values measured by a microplate reader (Figure 6B,C) showed little difference between the 1 × MIC and 3 × MIC treatments. As a result of its positive net charge and α-helix structure, AI-hemocidin 2 possesses bacterial membrane permeability activity. AI-hemocidin 2 functions in a similar manner to bactenecin, permeabilizing the bacterial membrane in a rapid, reliable way [49]. The results of our experiments suggested that AI-hemocidin 2 could increase the permeability of the cytoplasmic membrane of pathogens, thereby exerting antibacterial activity.

### 2.7. Propidium Iodide Uptake Experiment

**Figure 7:**
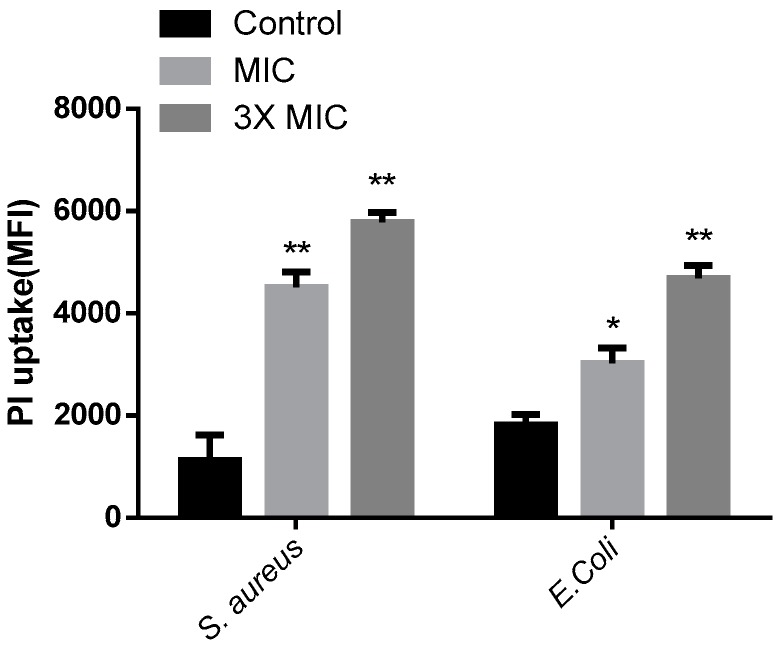
Effects of AI-hemocidin 2 on the membrane integrity of different bacterial strains. The data show the MFI (mean fluorescence intensity). * *p* < 0.05 and ** *p* < 0.01 versus control, and each assay was performed in triplicate. The results are presented as the mean ± SD.

Propidium iodide (PI), a DNA dye that passes through damaged cell membranes rather than integrating into the cell membrane, was used as a sensitive indicator to investigate cell membrane integrity and cell death by flow cytometry [50]. In the absence of peptide, the amount of PI uptake in *E. coli* and *S. aureus* cells was low, indicating an intact cell membrane. *E. coli* and *S. aureus* cells treated with 1 × MIC or 3 × MIC AI-hemocidin 2 displayed distinctly increased levels of PI uptake relative to cells incubated in the absence of AI-hemocidin 2 (Figure 7). A dose-dependent increase in PI fluorescence indicated that AI-hemocidin 2 was capable of damaging the *E. coli* and *S. aureus* cell membranes. Cell integrity damage assays were carried out concurrently with membrane permeabilization tests of antibacterial agents to establish whether these two effects correlated. For example, the proline-rich peptide Bac7(1-35) rapidly killed *E. coli* at micromolar concentrations, while PI remained outside the cells [50]. These results indicated that AI-hemocidin 2 was capable of damaging cell integrity and improving the permeability of the cell membrane.

### 2.8. Examination of the Morphologic Changes in Cells by Scanning Electron Microscopy

**Figure 8:**
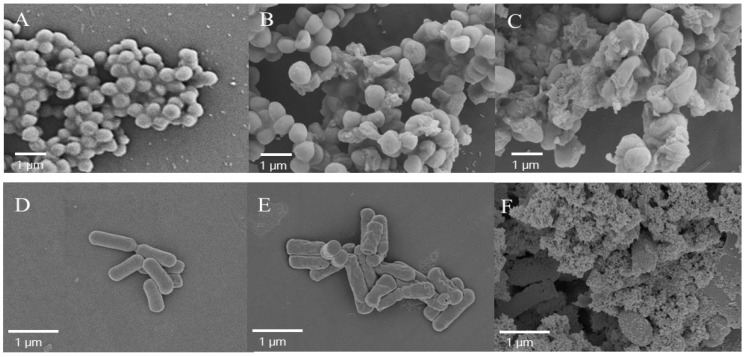
Scanning electron micrographs of *S. aureus* and *E. coli* treated with AI-hemocidin 2: (**A**) control, *S. aureus* without the peptide; (**B**) *S. aureus* treated with AI-hemocidin 2 at 1 × MIC for 1 h; (**C**) *S. aureus* treated with AI-hemocidin 2 at 1 × MIC for 3 h; (**D**) control, *E. coli* without peptide; (**E**) *E. coli* treated with AI-hemocidin 2 at 1 × MIC for 1 h; and (**F**) *E. coli* treated with AI-hemocidin at 1 × MIC for 3 h.

Direct visualization of AI-hemocidin 2-induced *E. coli* and *S. aureus* cellular damage was assessed by scanning electron microscopy (SEM). AI-hemocidin 2 treatment induced cell morphological changes. Compared with the control, 1 × MIC AI-hemocidin 2 treatment for 1 h induced partial damage of the *S. aureus* membrane, and severe membrane wrinkling and the release of cellular contents occurred within 3 h of treatment (Figure 8A–C).

The effects of AI-hemocidin 2 on *E. coli* are shown in Figure 8D–F. A smooth and intact membrane was exhibited in untreated *E. coli* cells. By contrast, membrane surface roughness and corrugation was observed after 1 h of treatment with 1 × MIC AI-hemocidin 2 (Figure 8E), and ruptured membranes and leakage of cellular contents were observed following 3 h of treatment with 1 × MIC AI-hemocidin 2 (Figure 8F). These results suggested that AI-hemocidin 2 is capable of destroying cellular integrity and inducing significant deformation in cellular morphology. The cell surface changes induced by AI-hemocidin 2 were similar to those previously reported [31,51]. Peptides interact with the cellular membrane via binding to negatively charged constituents on the outer membrane, such as lipopolysaccharide (LPS), which is followed by membrane destabilization [52]. In addition, it has previously been proposed that the α-helical conformation contributes to the antibacterial activity by disrupting membrane integrity [41].

## 3. Materials and Methods

### 3.1. Materials and Strain Culture Conditions

*A. inflata* materials were purchased from Chengyang seafood market, Qingdao, China, and were identified by Rongmin Yu (Jinan University, Guangzhou, China). The mantle mass was dissected, weighed and stored at −20 °C. DEAE Sepharose Fast Flow and SP Sepharose Fast Flow were purchased from GE Healthcare. Tris, SDS, Coomassie brilliant blue R-250, dimethyl sulfoxide (DMSO), PI, NPN, polymyxin B, ONPG, 3-(4,5-dimethyl-2-thiazolyl)-2,5-diphenyl-2*H*-tetrazolium bromide (MTT), streptomycin, and penicillin were obtained from Sigma Chemical Co. (St. Louis, MO, USA). RPMI-1640, fetal bovine serum (FBS) and LB broth were purchased from GIBCO Invitrogen Corporation (San Diego, CA, USA). Ciprofloxacin was purchased from Guangzhou Baiyunshan Pharmaceutical Co., Ltd. (Guangzhou, China). Other commercially available chemicals and reagents were of analytical grade.

Bacteria were grown in LB broth at 37 °C unless otherwise stated. LB medium was also used for antibacterial activity tests. All bacteria were stored as frozen cultures at −80 °C in appropriate culture medium with 30% (*v*/*v*) glycerol.

### 3.2. Screening for Antibacterial Activity Components

To rapidly screen the antimicrobial activity of each purified fraction, the disc diffusion method according to the National Committee for Clinical Laboratory Standards (NCCLS) was applied. *E. coli* was used as the test microorganism. A total of 10^6^ colony-forming units (CFUs)/mL of *E. coli* were poured onto solid LB medium plates. Afterwards, 20 μL of sample solutions (concentration of 2.00 mg/mL) was dropped onto sterilized filter paper (Ф = 6 mm) that had been pasted on the plates. The plates were completely dried before incubated at 37 °C for 24 h. The antibacterial activities of each purified fraction were determined by measuring the diameter of the inhibition zone with a vernier caliper. The fractions with a relatively large inhibition zone were used in subsequent studies. The experiments were performed in triplicate.

### 3.3. Purification of Hemoglobin Hb-I

Extraction and separation procedures were performed at 4 °C. *A. inflata* mantle mass (200 g) was completely homogenized (10,000 rpm, DS-1, Shanghai Jingke, Shanghai, China) and extracted using phosphate buffer (pH 8.0, 30 mM). The supernatant was precipitated with 70–100% saturation of solid ammonium sulfate then the precipitate was re-dissolved, dialyzed and lyophilized.

After lyophilization, the sample was applied to a diethyl-aminoethanol (DEAE)-sepharose Fast Flow anion exchange column (2.5 × 40 cm) with stepwise elute that had 0, 0.1, 0.3 and 2 M NaCl prepared in 30 mM Tris-HCl buffer (pH 8.0) at a flow rate of 1.0 mL/min. After purification by anion exchange chromatography, the fraction with the highest antibacterial activity was loaded onto a sulfopropyl (SP) Sepharose Fast Flow ion exchange chromatography column (2.5 × 40 cm) and stepwise eluted with increasing concentrations of NaCl (0, 0.5 and 1 M) dissolved in 30 mM phosphate buffer (pH 6.0). In each purification step, the elution was monitored at 280 nm by UV absorbance. Fractions or individual peaks were collected, dialyzed against distilled water using dialysis tube (MWCO 1000, Qiyun Biotech., Guangzhou, China) and freeze-dried for evaluation of the antibacterial activity.

### 3.4. Molecular Mass and Amino Acid Sequence Determination of Hemoglobin Hb-I

The precise molecular weight of the purified polypeptide was determined with an API type 4000 QTRAP mass spectrometer (Applied Bio system, Foster City, CA, USA), which was operated in positive electrospray ionization (ESI+ve) mode [33]. The gas used for drying (35 psi) and ESI nebulization (45 psi) was high-purity nitrogen. To determine the N-terminal amino acid sequence, the purified polypeptide was applied to a protein sequencer PROCISE 491 (Applied Biosystems, Foster City, CA, USA) and analyzed by Edman degradation according to a reported protocol [33]. The homology search of the N-terminal sequence was performed using BLAST software in NCBI.

### 3.5. Bioinformatics Analysis

Trypsin hydrolysis simulation of Hb-I was analyzed with the ExPASy Bioinformatics Resource Portal (http://www.expasy.org/tools/). Several trypsin-hydrolyzed peptides of Hb-I with potential antibacterial activity (AI-hemocidins) were discovered by screening with the APD database (http://aps.unmc.edu/AP/main.php). The physical and chemical parameters of AI-hemocidins were analyzed through the ExPASy Bioinformatics Resource Portal (http://www.expasy.org/tools/). The helix structures of the peptides were predicted by HeliQuest (http://heliquest.ipmc.cnrs.fr/).

### 3.6. Peptide Synthesis

AI-hemocidin 1 (PSVQGAAAQLTADVKK), AI-hemocidin 2 (DLRDSWKVIGSDKK), AI-hemocidin 3 (ISAAEFGKINGPIKK) and AI-hemocidin 4 (VLASKNFGDK) were synthesized by GL Biochem Ltd. (Shanghai, China) with solid-phase methods using *N*-(9-fluorenyl) methoxycarbonyl (Fmoc) chemistry. All peptides were analyzed by HPLC and mass spectrometry to confirm purity higher than 95%.

### 3.7. Determination of the Antibacterial Spectrum and Minimum Inhibitory Concentration

The antibacterial activity of Hb-I and AI-hemocidins was determined against both gram-positive and gram-negative bacteria strains, including *P. aeruginosa*, *E. coli*, *S. aureus*, *S. epidermidis*, *B. subtilis* and *E. faecalis*. Clinically isolated *P. aeruginosa* was provided by the First Affiliated Hospital of Jinan University. MICs of the samples were determined by a modified version of NCCLS broth microdilution method as reference [53]. The bacterial strains were inoculated and grown to mid-log phase in LB broth at 37 °C. Samples prepared in 0.9% sodium chloride solution were prepared in different concentrations with 2-fold serial dilution, from 0.5 to 1600 μg/mL. Ciprofloxacin was used as a control. A mixture of 90 μL of LB broth, 100 μL of inoculum suspension (1 × 10^6^ CFU/mL), and 10 μL of series concentrations for each sample was placed in sterile 96-well plates and incubated at 37 °C for 18 h. The absorbance at 600 nm was recorded with a microplate reader. The MIC was defined as the lowest concentration of the tested sample that completely prevented bacterial growth.

### 3.8. Hemolytic Assay and Cell Viability

Murine blood with citric acid treatment was centrifuged at 3000× *g* for 5 min, and the plasma and buffy coat were removed. Erythrocytes were washed three times with 0.9% saline solution and then re-suspended to 10 mM PB (pH 7.4) containing 150 mM NaCl. Red blood cells were then treated with serial dilutions of the peptide samples in a 96-well plate at 37 °C for 60 min. Control samples included a saline solution and 1% Triton X-100 for 0 and 100% hemolysis, respectively. Hemolysis was expressed as the hemoglobin content obtained from the absorbance of the supernatant at 570 nm after centrifugation at 3000× *g* for 5 min [53,54]. The hemolysis assay was performed in triplicate.

Human HEK293 embryonic kidney cells (1 × 10^5^ cells/mL) were incubated in 96-well plates using Dulbecco’s modified Eagle’s medium (DMEM) that contained 10% fetal calf serum, penicillin (100 U/mL) and streptomycin (100 μg/mL) at 37 °C in a humidified incubator with 5% CO_2_ atmosphere. Cell viability was evaluated by conventional MTT reduction assays [53]. The absorbance at 570 nm of the resulting solution was measured with a microplate reader (Synergy 2, Biotech, VT, USA). The experiments were performed in triplicate.

### 3.9. Effects of pH and Temperature on the Antibacterial Activity

To investigate the effects of the temperature and pH on the antimicrobial activities of AI-hemocidin 2, the disc diffusion method was applied against *S. aureus*, *P. aeruginosa* and *E. coli* [55]. Samples were incubated with the following solutions: 0.01% NaOH (pH 10), 0.01% HAc (pH 3) and sterile water (pH 7). After 1 h of incubation in the appropriate solution, the sample solutions were determined using the microdilution method. To determine the thermal stability, samples were incubated at 100 °C for 10 min. Then, the samples were cooled and used for determination. Each assay was performed in triplicate.

### 3.10. Membrane Permeability Assay

To assess the cell membrane permeability ability of AI-hemocidin 2, the membrane permeability assay was applied with *E. coli* and *S. aureus*. The outer membrane permeability assay for *E. coli* was performed using NPN uptake assay [56]. *E. coli* were cultured to the logarithmic phase in LB broth at 37 °C and adjusted to an OD_600_ of 0.5. Cells were washed 3 times and re-suspended with 5 mM HEPES buffer (pH 7.2). NPN was added to 1 mL of cells in a quartz cuvette with a final concentration of 10 µM. After 180 s, the peptide samples at their 1 × MIC and 3 × MIC were added to the cuvette. The fluorescence increase by partitioning NPN into the outer membrane was measured using a fluorescence spectrometer (HITACHI F-4500, HITACHI, Tokyo, Japan) until there was no further increase in fluorescence. The values were recorded at 360 s after sample treatment. The outer membrane permeability was evaluated by the % NPN uptake.

% NPN uptake = (Fobs − F0)/(F100 − F0) × 100(1)

where Fobs is the observed fluorescence at a given peptide concentration, F0 is the initial fluorescence of NPN with bacteria in the absence of peptide, and F100 is the fluorescence of NPN with bacteria cells upon the addition of 10 µg/mL Polymyxin B, a positive control with a strong outer membrane permeabilizing property.

The permeabilities of the *E. coli* inner cell membrane and *S. aureus* cell membrane were assessed by detection of ONPG as a substrate accounting for its non-membrane permeation. *E. coli* and *S. aureus* were incubated to the mid-log phase, washed, and re-suspended in 10 mM PBS (pH 7.4) [57]. ONPG (1.5 mM) was dissolved in the same buffer, and peptide samples at their 1 × MIC and 3 × MIC were incubated with microbial cells. The hydrolysis of ONPG to o-nitrophenol was monitored at 405 nm every 30 min using a microplate reader. A cell suspension without sample was used as a control. 

### 3.11. Integrity of the Bacterial Membrane

The bacterial membrane integrity was evaluated by determining the uptake of PI using BD Accuri C6 flow cytometry (Ann Arbor, MI, USA) [58]. Tested bacterial strains (*E. coli* and *S. aureus*) were incubated to mid-log phase in LB broth at 37 °C. The density of the bacterial suspensions was adjusted to 1 × 10^6^ CFU/mL by dilution in LB broth containing AI-hemocidin 1 and AI-hemocidin 2. The bacterial suspension was incubated at 37 °C for 60 min with different samples at the MIC or 3 × MIC. PBS (10 Mm, pH 7.4) was used as a negative control. Afterwards, sample-treated bacteria were washed twice and re-suspended with PBS. Then, the suspensions were incubated with PI (100 μM) for 10 min in an ice bath. In flow cytometry, 40,000 events were collected for each sample at the medium flow rate with an excitation and emission at 488 and 525 nm, respectively.

### 3.12. Examination of Bacterial Membrane Damage by SEM

*E. coli*, and *S. aureus* were cultured to the logarithmic phase in LB broth and then harvested by centrifugation at 1000× *g* for 10 min. The cells were washed 3 times with 10 mM PBS (pH 7.4) and re-suspended to an OD_600_ of 0.2. Thereafter, the bacterial suspension was incubated at 37 °C for 1 or 3 h with different samples at 1 × MIC. Following incubation, the cells were collected and washed 3 times with 10 mM PBS at 3000× *g* for 5 min. Bacterial cell pellets were then fixed overnight with 2.5% (*v*/*v*) glutaraldehyde in PBS at 4 °C and then washed twice with PBS. Afterwards, the cell pellets were dehydrated in a graded ethanol series (50, 60, 70, 80, 90 and 100%) for 10 min each. The dried samples were transferred to a mixture (1:1, *v*/*v*) of ethanol and isoamyl acetate for 20 min. The specimens were dried using a critical point dryer that was coated with gold. The cell morphology was observed under a field emission SEM (Carl Zeiss Ultra 55, Oberkochen, Germany) [44].

### 3.13. CD Determination and Secondary Structure Prediction

The secondary structures of AI-hemocidin 2 in mimicking aqueous environment and mimicking the hydrophobic environment of the microbial membrane conditions were measured using a J-820 spectropolarimeter (Jasco, Tokyo, Japan) [43]. The spectra were recorded at a scanning speed of 10 nm/min at wavelengths range from 195 to 250 nm in 10 mM PBS and 50% TFE. All procedures were performed at 25 °C, and an average of three scans was collected for each sample. The final sample concentration was 150 μM. The secondary structure of AI-hemocidin 2 was built by homology modeling using the I-TASSER server (http://zhanglab.ccmb.med.umich.edu/I-TASSER/).

### 3.14. Statistical Analysis

The software package SPSS Statistics 20.0 (SPSS Inc., Chicago IL, USA) for Windows was used for statistical analysis and GraphPad Prism 5.0 (GraphPad Software, San Diego, CA, USA) was used for the presentation of data. The experimental data are presented as the mean ± standard deviation (SD) unless otherwise indicated. SPSS Statistics was used to calculate values according to one way analysis of variance (ANOVA) followed by Duncan’s multiple range tests. The independent two-sample Student’s t-test was used to establish significant differences in the experiment of the outer membrane permeabilization of *E. coli* by AI-hemocidin 2. *p* values of less 0.05 were considered to be significantly different.

## 4. Conclusions

In this study, an antibacterial hemoglobin subunit Hb-I was purified from the mantle mass of *A. inflata*. A novel N-terminal peptide of hemoglobin, AI-hemocidin 2, was identified from Hb-I by bioinformatic analysis. AI-hemocidin 2 exhibited broad-spectrum antibacterial effects against Gram-negative and Gram-positive bacteria and showed a typical α-helical structure in the membrane mimetic environment. AI-hemocidin 2 also displayed low hydrolysis activity and cytotoxicity with bacterial selectivity. The results of membrane permeabilization, flow cytometry and SEM assays demonstrated that one of the mechanisms of AI-hemocidin 2-induced cell death might be to disrupt the bacterial membrane. These findings revealed that AI-hemocidin 2 might be an attractive and promising candidate as a nature-derived antibacterial agent against pathogenic bacteria. In addition, exploring the peptide fragments of hemoglobin by employing bioinformatic analysis is a feasible approach for identifying antibacterial peptides.

## Figures and Tables

**Table 1:** Antibacterial activity of each purified fraction against *E. coli* ATCC25922.

Fractions	J1	J2	J3	J4	J1S1	J1S2	J1S3	Cipro
IZ (mm)	10.18 ± 0.24	9.22 ± 0.21	nd	nd	16.19 ± 0.62	nd	6.74 ± 0.18	31.66 ± 0.22

IZ = Inhibition zone (mm). nd = not detected. Cipro = Ciprofloxacin. Sample amount: 20 μL. The concentration of samples was 2.00 mg/mL. Each assay was performed in triplicate, and the results are presented as the mean ± SD.

**Table 2:** Sequences and physicochemical properties of AI-hemocidins.

Peptide	Sequence	Length	pI	Mw	NC	H	PR/n %
1	PSVQGAAAQLTADVKK	16	9.01	1583.81	1	0.196	8/50%
2	DLRDSWKVIGSDKK	14	8.50	1646.86	1	0.043	10/71.43%
3	ISAAEFGKINGPIKK	15	9.70	1572.87	2	0.285	8/53.33%
4	VLASKNFGDK	10	8.56	1078.23	1	0.163	6/60%

pI: isoelectric point. Mw: molecular weight. NC: net charge. H: hydrophobicity. PR/n %: polar residues and ratio of polar residues.

**Table 3:** Antibacterial activities of Hb-I and AI-hemocidins.

Bacteria	MIC Value (μM)					
	1	2	3	4	Hb-I	Cipro
*E. coli* ATCC 25922	47.35	45.54	>381.47	>556.47	75.66	24.14
*P. aeruginosa* ATCC 27853	47.35	91.08	95.37	>556.47	>100.88	48.28
*P. aeruginosa* clinical isolate	47.35	91.08	>381.47	>556.47	>100.88	48.28
*S. aureus* ATCC 25923	>378.83	22.77	>381.47	>556.47	75.66	24.14
*B. subtilis* ATCC 6633	>378.83	182.16	>381.47	69.55	>100.88	12.07
*B. subtilis* ATCC 6633	>378.83	182.16	>381.47	>556.47	>100.88	12.07
*E. faecalis* ATCC 29212	>378.83	>364.33	>381.47	>556.47	>100.88	24.14

Each assay was performed in triplicate. Cipro = Ciprofloxacin.

**Table 4:** Hemolysis activity and cytotoxicity of AI-hemocidin 2.

Sample	AI-Hemocidin-2
MIC ^a^ (μg/mL)	37.5–300
HC_10_^b^ (μg/mL)	>500
H_max_^c^	10.27 ± 0.42
Cell viability (%) at 250 μg/mL	88.18 ± 9.48
IC_50_^d^(μg/mL)	>1000
SI value ^e^	3.33–26.67

Note: ^a^ Minimum inhibitory concentration of AI-hemocidin 2 against *E. coli*, *P. aeruginosa*, *P. aeruginosa* clinical isolate, *S. aureus*, *S. epidermidis* and *B. subtilis*. ^b^ HC_10_ is the concentration of peptide causing 10% hemolysis on human erythrocytes. ^c^ H_max_ is the percentage (%) hemolysis at the highest tested peptide concentration (500 μg/mL). ^d^ IC_50_ is defined as the concentration at which 50% of growth is inhibited. ^e^ Selectivity index (in vitro): IC_50_ in HEK 293 cells/MIC, IC_50_ taken as a minimum, 1000 μg/mL. Assays were performed in triplicate, and the results are presented as the mean ± SD.

## Supplementary Materials

The following are available online at www.mdpi.com/1660-3397/15/7/205/s1, Figure S1: Purity confirmation of J1S1. (A) The result of Tricine-SDS-PAGE; (B) The result of Native PAGE.; Figure S2: The result of n-terminal amino acid sequencing of Hb-I.
