# Supplementary material for: Antibacterial Activity of AI-Hemocidin 2, a Novel N-Terminal Peptide of Hemoglobin Purified from Arca inflata
Source: Mar Drugs. 2017 Jun 29;15(7):205. doi: 10.3390/md15070205 (PMC5532647; doi:10.3390/md15070205)
Supplement: Supplementary file 1 [file marinedrugs-15-00205-s001.pdf]

Supplementary Materials: Antibacterial Activity of AI-Hemocidin 2, a Novel N-Terminal Peptide of Hemoglobin Purified from *Arca inflate*

Chunlei Li [1,+], Jianhua Zhu [1,+], Yanqing Wang [2,+], Yuyan Chen [1], Liyan Song [3], Weiming Zheng [3], Jingjing Li [3] and Rongmin Yu [1,3,*]

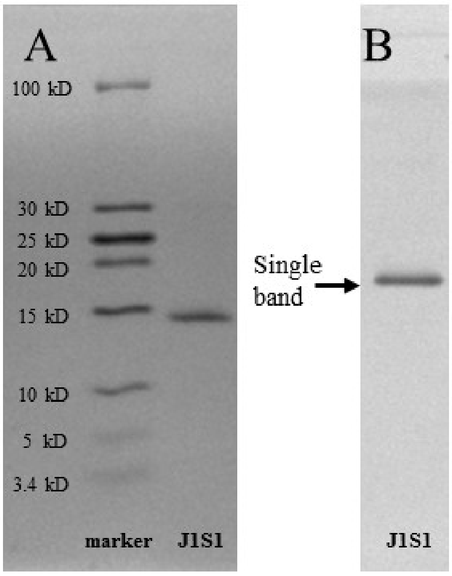

Figure S1. Purity confirmation of J1S1. (**A**) The result of Tricine-SDS-PAGE; (**B**) The result of Native PAGE.

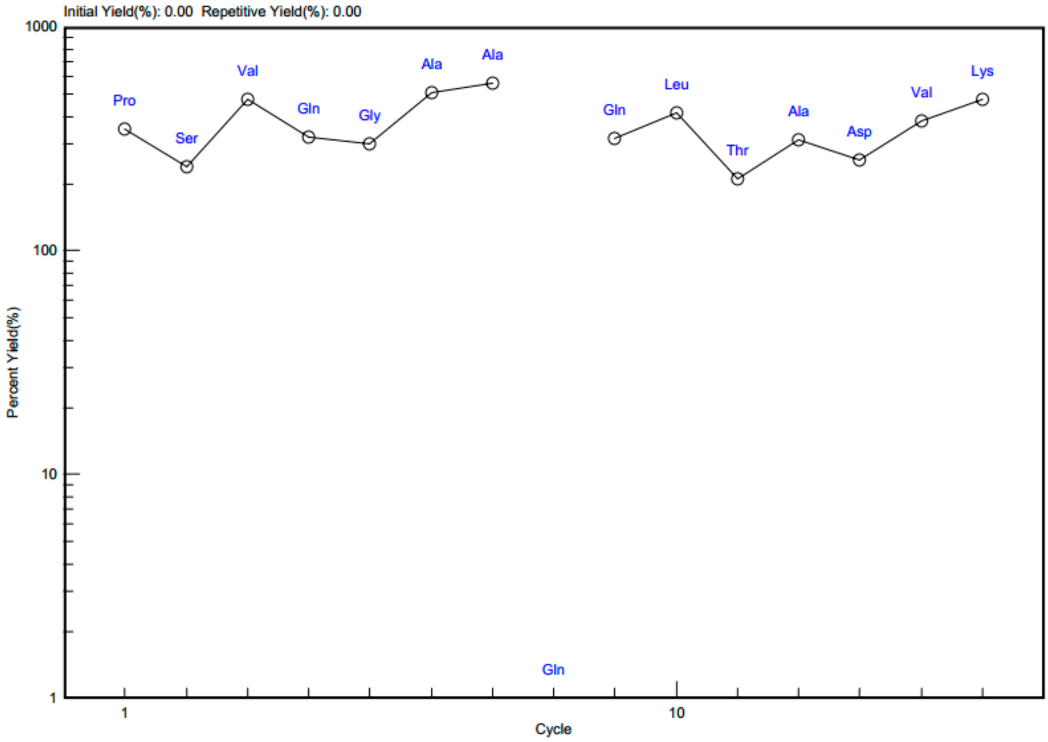
Initial Yield(%): 0.00 Repetitive Yield(%): 0.00
Percent Yield(%)
Cycle
1000
100
10
1
1
10
Pro
Ser
Val
Gln
Gly
Ala
Ala
Gln
Gln
Leu
Thr
Ala
Asp
Val
Lys

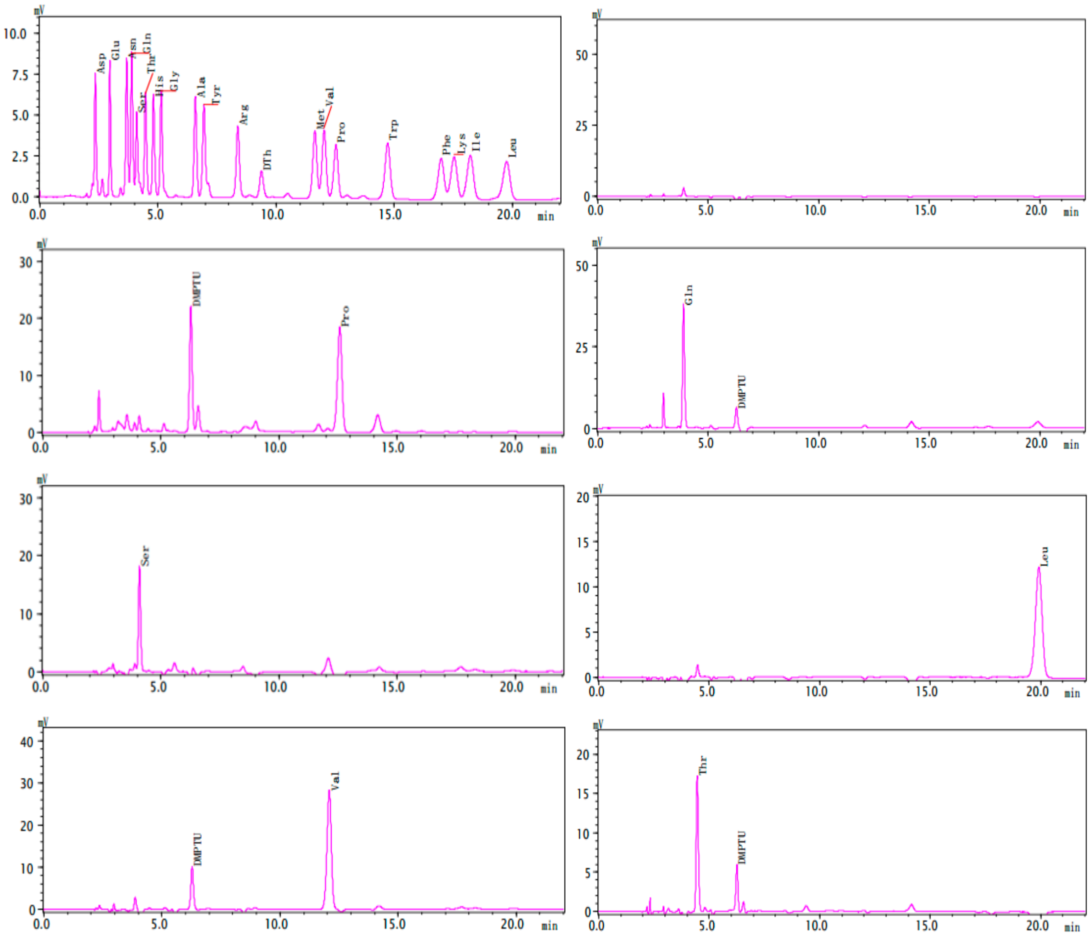
mV
min
Asp
Glu
Asn
Gln
Ser
Thr
His
Gly
Ala
Tyr
Arg
DTh
Met
Val
Pro
Trp
Phe
Lys
Ile
Leu
DMPTU
Pro
Gln
DMPTU
Ser
Leu
DMPTU
Val
Thr
DMPTU

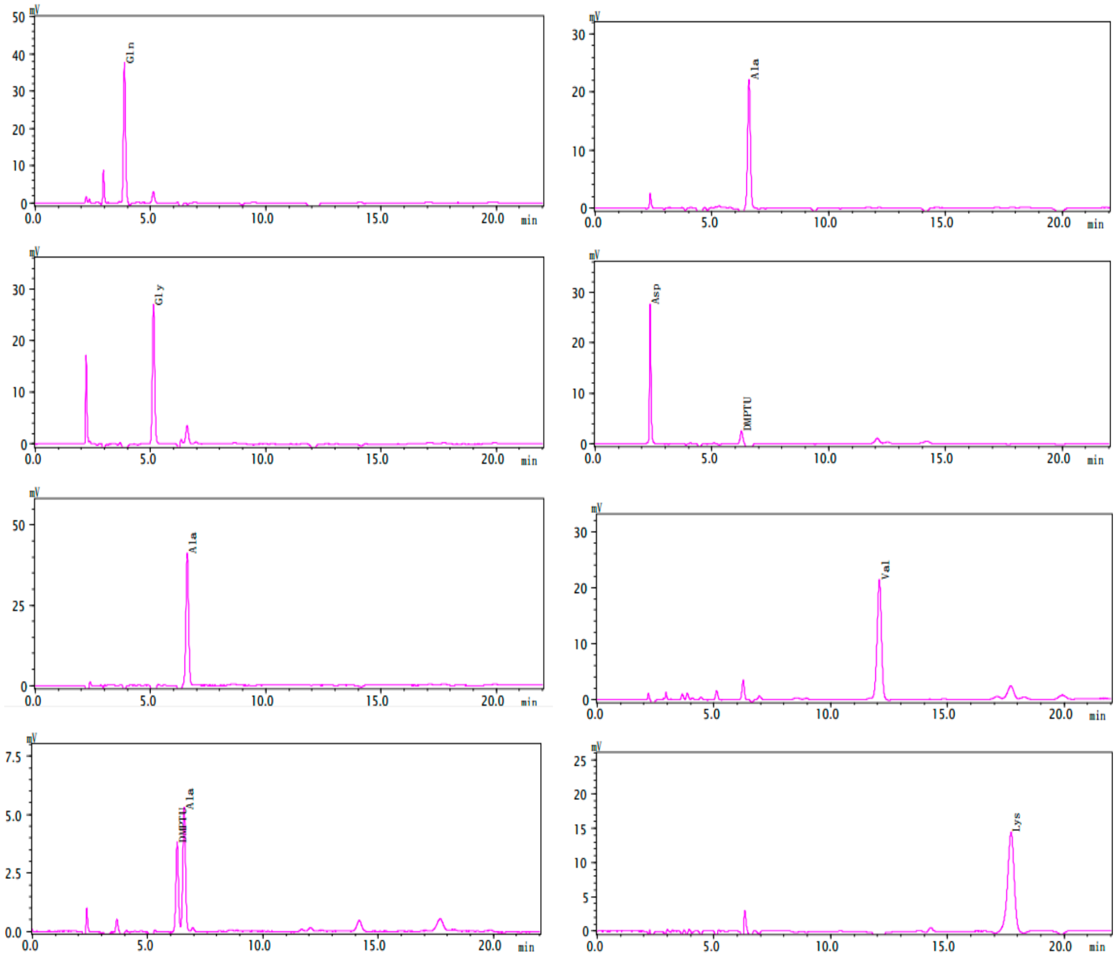

Figure S2. Results of N-terminal amino acid sequencing of Hb-I.
